# Consent in emergency psychiatry : a literature review

**DOI:** 10.1192/j.eurpsy.2023.1829

**Published:** 2023-07-19

**Authors:** I. Katir, A. Korchi, Z. Bencharfa, S. Belbachir, A. Ouanass

**Affiliations:** Psychiatry, Psychiatric Hospital Ar-razi of Salé, Salé, Morocco

## Abstract

**Introduction:**

The fundamental principle of medical ethics is based on the principle of autonomy, of which consent is part, in addition to the right to information and the free choice of caregiver by the patient. In the psychiatric emergency department, the psychiatrist is confronted in his daily practice with the decisions of outpatient or hospital care, sometimes without the consent of the patients, in particular when the disorders hinder their capacities for self-assessment and judgment or when he there is a vital prognosis involved.

**Objectives:**

It is therefore important to know certain basic legal rules in order to better manage these emergency situations.

**Methods:**

Literature review

**Results:**

Patients requiring care in the emergency department present particular challenges to ensuring valid consent. Patients often present in a crisis situation and their abilities may be altered by, for example, psychoactive substances or impaired judgment. These patients are cared for by clinicians who may have to make urgent decisions based on incomplete information. The emergency department can be a disorienting and frightening environment for patients. The following aspects of consent: autonomy and capacity, are particularly relevant to care in emergency departments.

**Conclusions:**

The interplay between emergent need, presumed incompetence, implied consent, and societal interest, as well as the individual details of each case, are all important in making the right ethical and legal decision in an emergency situation. given, because the emergency situation does not allow any delay in the decision-making process.

**Disclosure of Interest:**

None Declared

